# Ascending cholangitis in gastric bypass patients following hepatobiliary scintigraphy and oral protein shake administration: a case report

**DOI:** 10.1093/jscr/rjaf365

**Published:** 2025-06-06

**Authors:** Mahmoud K Abd-El-Hafez, Monte L Roper, Kevin Durkee, Roger A De la Torre

**Affiliations:** Menorah Medical Center Department of General Surgery Residency, HCA Midwest Healthcare System, Overland Park, KS 66209, United States; Menorah Medical Center Department of General Surgery Residency, HCA Midwest Healthcare System, Overland Park, KS 66209, United States; Department of Bariatric and Metabolic Surgery, HCA Midwest Healthcare System, Overland Park, KS 66209, United States; Department of Bariatric and Metabolic Surgery, HCA Midwest Healthcare System, Overland Park, KS 66209, United States

**Keywords:** ascending cholangitis, HIDA scintigraphy scan, CCK administration, Roux-en-Y gastric bypass, biliary balloon angioplasty, transcystic CBD exploration

## Abstract

We present the unusual case of a 66-year-old female who was found to have ascending cholangitis following hepatobiliary scintigraphy and fatty meal administration, in the setting of isolated cholelithiasis. Given her surgical history of Roux-en-Y gastric bypass, a robotic cholecystectomy with transcystic common bile duct (CBD) exploration was performed. Patient was discharged on post-operative day (POD) 7 with a T-tube following antibiotic completion. Tube study at 1 week confirmed resolution of her CBD outlet obstruction. Acute cholangitis is a concerning outcome following hepatobiliary iminodiacetic acid scan and cholecystokinin (CCK) administration in patients with preexisting cholelithiasis. We do believe that this is a risk that warrants consideration and informed patient discussion when using this imaging modality. We also present an early experience with transcystic common bile duct exploration with balloon dilation. A technique, we believe, will be of benefit for our bariatric patient population when presenting with choledocholithiasis or acute cholangitis.

## Introduction

Hepatobiliary iminodiacetic acid (HIDA) scan is an imaging modality frequently used to diagnose both acute cholecystitis and biliary dyskinesia. One, as yet-to-be-demonstrated, complication of the HIDA scan is the possibility for stone migration during induction of gallbladder contraction. This can lead to choledocholithiasis or acute cholangitis. To our knowledge, a case of HIDA induced choledocholithiasis has never been presented. Furthermore, we present our early experience with transcystic common bile duct exploration with balloon dilation in this bariatric patient.

## Case report

66-year-old female presented to the emergency department for a 1-month history of worsening postprandial pain and distention. Physical exam was unremarkable except for tenderness to palpation in the upper quadrants. Medical history included hypertension, hyperlipidemia, DM2, RA, and hyperparathyroidism. Past surgical history most notable for a Roux-en-Y gastric bypass, ventral hernia w/mesh, and reduction of internal hernia.

Baseline medications were Atorvastatin, Infliximab, Pantoprazole, and multimodal pain control. Pertinent lab findings included white blood count (WBC) 7.9 (4.1–11.1), Hg 12.7 (11.5–15.3), Total Bilirubin 0.4 (0.0–1.0), AST 30 (10–45), ALT 69 (12–78), Lipase 29 (13–77).

**Figure 1:**
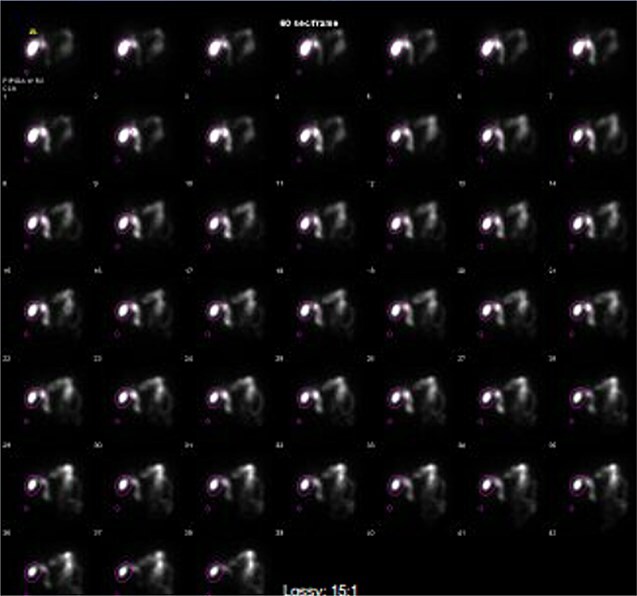
HIDA scan showing normal gallbladder filling and rapid emptying of nucleotide contrast in the SB following administration of CCK.

Computed tomography (CT) in the ER showed normal gastric bypass anatomy with moderate colonic stool burden. Negative for biliary pathology. Follow up ultrasound showed cholelithiasis without evidence of acute cholecystitis. A confirmatory HIDA showed normal filling of the gallbladder with rapid clearance into the small bowel and ejection fraction of 62% (Fig. 1).

Two days later, patient reported new-onset chills/shakes. By the following morning patient was in obvious sepsis. Vitals at that time was BP 91/57 and temp 103.1°F. Lab findings were WBC 19.3 (4.1–11.1), aspartate aminotransferase (AST) 1144 (10–45), alanine transaminase (ALT) 1339 (12–78), total bilirubin 1.3 (0.0–1.0), lipase 218 (13–77).

**Figure 2:**
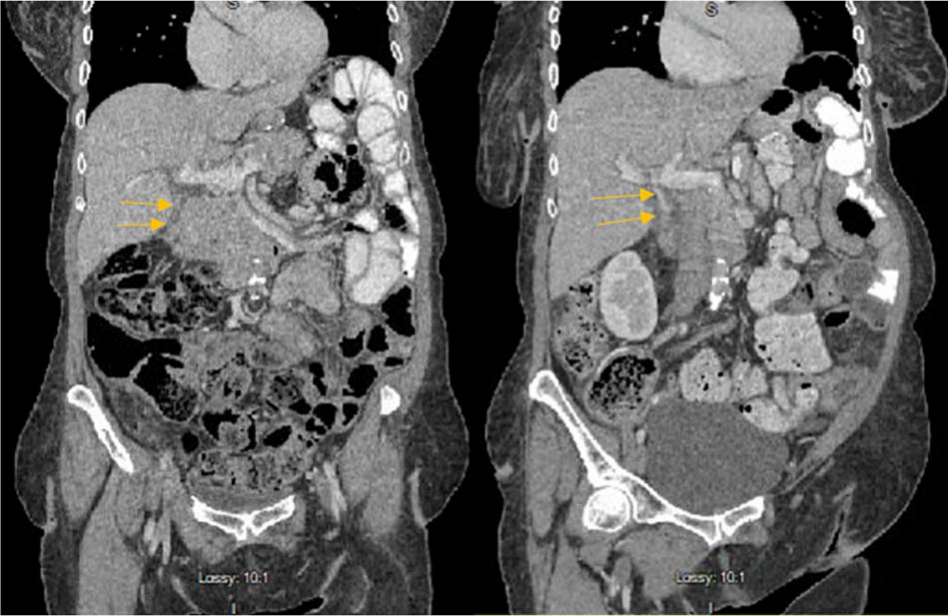
Hyperdense material (arrows) found in the CBD on the post-HIDA CT (right) that was not previously seen on the pre-HIDA CT on admission (left).

**Figure 3:**
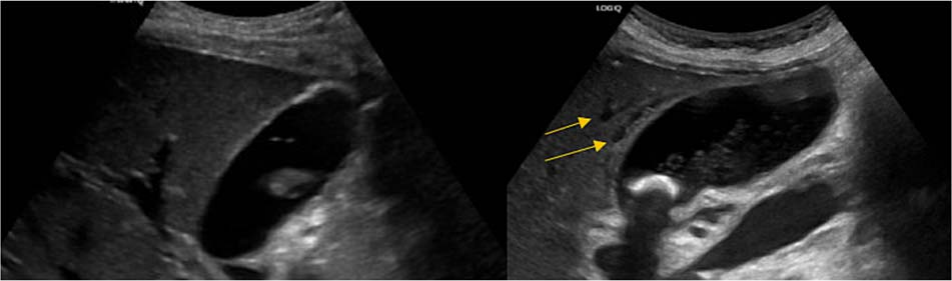
Pericholecystic fluid (arrows) and positive sonographic murphy sign present on the post-HIDA ultrasound (right) that was not previously seen on the pre-HIDA ultrasound on admission (left).

Stat CT scan showed new onset pericholecystic edema and hyperdense material within the common bile duct (CBD), not previously seen (Fig. 2). Stat Ultrasound confirmed findings of new onset acute cholecystitis (Fig. 3). Given the patient’s surgical history of gastric bypass, the decision was made to proceed with a robotic cholecystectomy with transcystic common bile duct exploration.

**Figure 4:**
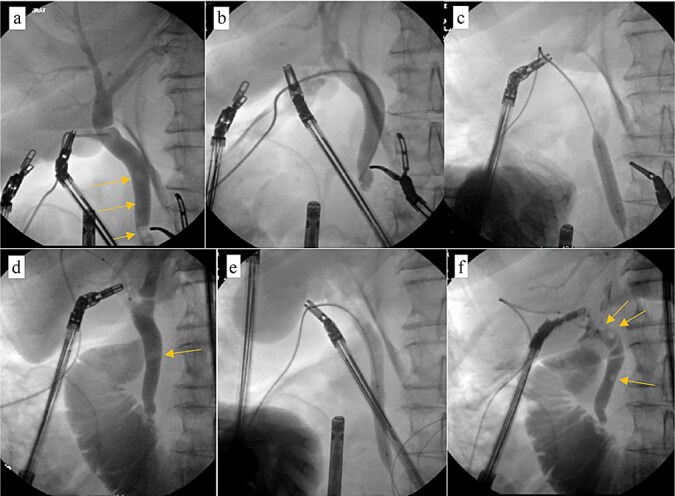
Intraoperative cholangiogram. (a) Initial cholangiogram via 6-fr ureteral stent showing multiple filling defects (arrows). (b) Guidewire passed through the ureteral stent and down to the level of the ampulla of vater, demonstrating complete CBD obstruction. (c) 7 × 40 mm vascular catheter balloon advanced over the guidewire and through the ampulla of vater. Dilated to nominal pressure. (d) Contrast now able to leave the CBD and into the duodenum. Residual “floating” filling defects still seen (arrows). (e) Vascular catheter exchanged for a 9 × 40 mm balloon and dilated to nominal pressure. (f) Completion cholangiogram shows brisk outflow of contrast with residual filling defects (arrows).

Patient was brought to the OR, induced, and the peritoneum was accessed via optical view trocar. After the critical view of safety was obtained, the cystic duct was partially transected and a 6-French ureteral catheter was threaded through the duct and into the CBD. Initial cholangiogram showed complete obstruction of the distal CBD. A 0.035 guidewire was advanced through the ureteral catheter. The ureteral catheter was then exchanged for a 9 × 40 mm long vascular balloon catheter. The balloon was then centered at the ampulla of vater and inflated to its nominal pressure for 5 min. A small indentation was noticed at the level of the ampulla once the balloon was fully insufflated, as expected. The completion cholangiogram showed prompt evacuation of contrast into the duodenum. However, numerous “floating” filling defects continued to be present (Fig. 4). The decision was thus made to leave a T-tube in place via a 1-cm longitudinal choledochotomy. The incision was reapproximated around the t-tube with interrupted 4–0 PDS. The vascular balloon catheter was removed, the cystic duct was completely transected, and the gallbladder was dissected off the liver bed.

**Figure 5:**
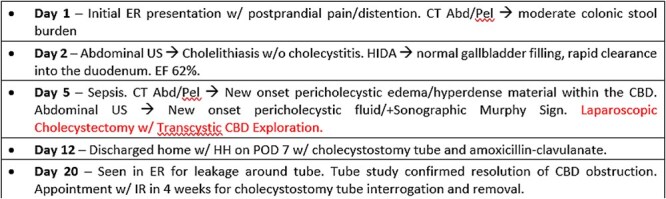
Timeline of events from initial ER presentation to discharge following postoperative recovery.

**Figure 6:**
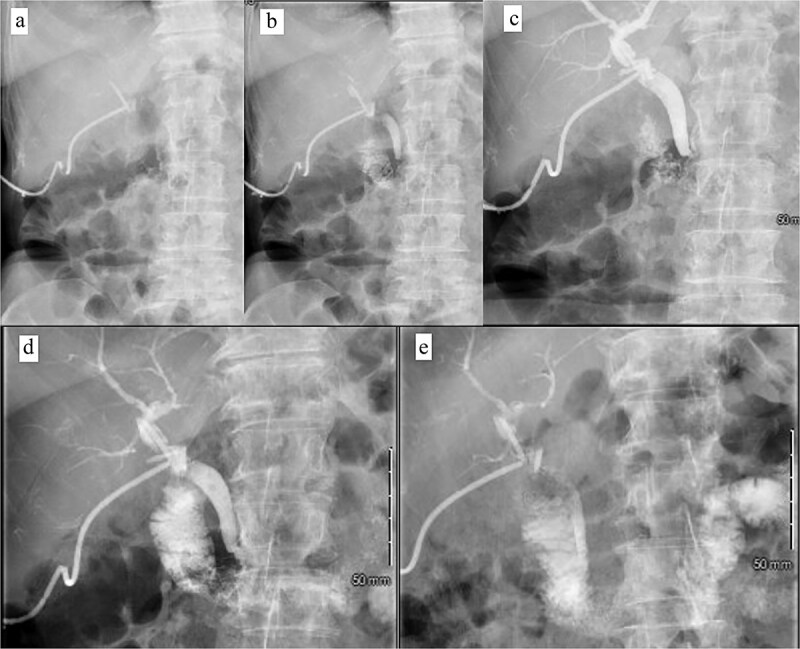
Postoperative tube study in ER. (a) Contrast injected into the cholecystostomy tube. (b) Contrast entering the CBD. (c) Contrast completely filling the CBD and intrahepatic ducts. (d) Contrast leaving the CBD and into the duodenum. (e) Contrast predominately in the small bowel.

Patient was discharged home on POD 7 following IV antibiotic completion (Fig. 5). Patient was seen in the ER 1 week later for drainage around the T-tube. Tube study confirmed resolution of CBD obstruction and the patient was discharged from the ER (Fig. 6).

## Discussion

Hepatobiliary iminodiacetic acid (HIDA) scans are useful imaging techniques for diagnostic elucidation of acute cholecystitis and biliary dyskinesia when the clinical picture is unclear. The HIDA scan is performed by injecting a radiotracer (iminodiacetic acid) intravenously, which then preferentially collects in the liver where it is secreted into the ductal system. The ductal system is then able to be visualized along with the patency of the cystic duct and the functionality of the sphincter of Oddi [1]. After this initial test, either cholecystokinin or a “fatty meal” (often a protein shake) can be administered in order to induce gallbladder contractions. This allows for the evaluation of the gallbladder clearance as well as the overall gallbladder functionality by calculating the “ejection fraction (EF)” [2].

Our patient did not show radiographic evidence of choledocholithiasis prior to undergoing her HIDA scan. The HIDA scan itself was negative for cholecystitis and showed a normal EF (62%). The patient then had migration of debris into her CBD in the following 48 h. It is hypothesized that the HIDA scan was the inciting factor for the development of this patient’s ascending cholangitis.

This patient underwent a fatty meal administration during her HIDA in order to facilitate gallbladder contraction. This was the theorized catalyst for stone migration. While some baseline risk would have been assumed with any fatty food administration regardless of its association with the HIDA scan, it is not generally discussed with patients in the setting of HIDA scan or cholecystokinin (CCK) administration [3]. This patient’s situation was further complicated by her history of gastric bypass, which precluded the ability to do a traditional ERCP.

This case report also outlines early experience in our institution with transcystic common bile duct exploration with antegrade balloon sphincteroplasty as outlined by Bosley *et al.* [4] This technique represents a significant potential for patient populations that have anatomic alterations that preclude them from getting an ERCP, such as in the bariatric surgery population. In this particular case, there was some struggle with sphincter of Oddi spasm, likely due to insufficient balloon inflation up-time and CBD/Vascular balloon diameter mismatch. Further investigation into this particular modality is certainly warranted.

In conclusion, we report a case of a post-HIDA ascending cholangitis that resulted from stone migration during fatty food oral administration. We believe that this case will serve as an example to the various etiologies of acute cholangitis. This case report also raises awareness of a poorly communicated potential complication which every patient should be made aware of prior to undergoing this imaging modality. Furthermore, this case is an example of an expanding technique for transcystic common bile duct exploration in the bariatric patient population.
